# Management of Post-Traumatic Pseudomeningocele as Consequence of Root Nerve Avulsion: Case Report and Review of the Literature

**DOI:** 10.3390/neurolint16060126

**Published:** 2024-12-06

**Authors:** Leonardo Bradaschia, Filippo Lacatena, Francesca Vincitorio, Paolo Titolo, Bruno Battiston, Diego Garbossa, Fabio Cofano

**Affiliations:** 1 Department of Neuroscience “Rita Levi Montalcini”, University of Turin, 10126 Turin, Italy; leonardo.bradaschia@unito.it (L.B.); filippo.lacatena@unito.it (F.L.); diego.garbossa@unito.it (D.G.); fabio.cofano@unito.it (F.C.); 2 Neurosurgery Unit, Department of Neuroscience “Rita Levi Montalcini”, A.O.U. Città della Salute e della Scienza Torino University of Turin, 10126 Turin, Italy; vincitorio.francesca@gmail.com; 3 Hand and Upper Limb Surgery Unit, Department of Orthopedic and Traumatology, Orthopedic and Trauma Center, Molinette Hospital, Città della Salute e della Scienza, 10126 Turin, Italy; paolo.titolo@unito.it

**Keywords:** root nerve avulsion, pseudomeningocele, traumatic brachial plexus, management, external lumbar drainage

## Abstract

Background: Post-traumatic pseudomeningoceles are common findings after a brachial or lumbar plexus trauma, in particular after nerve root avulsion. Unlike meningoceles, pseudomeningoceles are CSF full-filled cysts confined by the paraspinous soft tissue, along the normal nerve course, in communication with the spinal subarachnoid spaces. Normally no more than a radiological finding at MRI, in rare instances they might be symptomatic due to their size or might constitute an obstacle during a reconstructive surgery. Methods: A review of the literature was performed in accordance with the Preferred Reporting Items for Systematic Reviews and Meta-Analyses (PRISMA) guidelines in a time span ranging from November 1972 to May 2024. A total of five articles were found meeting the inclusion criteria. A case report at our institution was added to the case history. Results: A 30-year-old man with complete right brachial plexus nerve roots avulsion and a voluminous pseudomeningocele at the C6-C7 level after a motorcycle incident in January 2023. The pseudomeningocele covered the entirety of the injured brachial plexus. Pre-operative external lumbar drainage was utilized to prevent relapse or worsening of the already existing cerebral spinal fluid collection, with good results at 6 months. The full case report is reported in detail. Conclusions: To date, no clear guidelines about the management of post-traumatic pseudomeningoceles are reported in the literature. The lack of symptoms or signs related to them does not usually require any surgical intervention. If not, a possible management strategy with the use of an external lumbar drainage is proposed, a solution already in use in other surgical contexts with successful results in preventing CSF fistula or its relapse.

## 1. Introduction

Common findings after a brachial or lumbar plexus trauma, post-traumatic pseudomeningoceles are normally no more than a radiological finding at Magnetic Resonance Imaging (MRI). Precisely, they are extradural collections of Cerebral Spinal Fluid (CSF) that result following a breach in the dural–arachnoid layer; they differentiate from a true meningocele by the lack of an arachnoid-lined capsule [1]. The majority of pseudomeningoceles are iatrogenic, resulting from incidental durotomies during spinal or intradural surgery [2], while in rare circumstances they might originate after a high energy trauma, in particular in association with nerve roots avulsion. In the last case, damaged meninges surrounding damaged nerve roots are the source of the CSF leakage, with its collection in the soft tissue and the formation of the pseudomeningocele. Their true incidence is not known, as there are few cases reported in the literature [3].

Although most of the time they do not need any surgical intervention and are completely asymptomatic, in exceedingly rare occasions they can exert a mass effect over time with compression of the near nervous structures, cause an intracranial hypotension, or hinder a surgical operation; a management strategy is therefore required. The main scope of this review is to investigate whether a surgical strategy for the management of post-traumatic pseudomeningoceles secondary to nerve root avulsion is present in the literature, both for their repair and to avoid CSF leakage after surgery.

## 2. Materials and Methods

The review was performed in accordance with the Preferred Reporting Items for Systematic Reviews and Meta-Analyses (PRISMA) guidelines [4]. Furthermore, a single case-report reported at our institution was added to the case history.

### 2.1. Literature Search

All full-text English-language manuscripts on the management of post-traumatic pseudomeningocele were screened using the PubMed/MEDLINE, Embase, Cochrane Library, Scopus, and Web of Science databases, in a time span going from November 1972 to May 2024. Search terms included code words or their combinations with associated Boolean operators, such as “Post traumatic” AND “Pseudomeningocele” AND “Roots nerve avulsion” OR “Brachial plexus” OR “Lumbar plexus” OR “Management” OR “Treatment”.

### 2.2. Studies Selection

The rough list of articles was screened on the basis of title, abstract, and full text for the potential to meet the eligibility criteria, and any discrepancies were resolved by consensus. Studies were included if the following predefined inclusion criteria were met: case reports written in English, cases referring to the management and/or treatment of post-traumatic pseudomeningocele in brachial/lumbar plexus injuries, and reporting at least one outcome of interest (i.e., it was not mandatory that all outcomes of interest were reported in the study). Articles regarding the management of post-traumatic retropharyngeal pseudomeningocele secondary to an Atlanto-Occipital Dislocation (AOD) were excluded since the pathophysiology and mechanisms of the pseudomeningocele formation differ from a nerve root avulsion, involving high-energy trauma of the cervical spine but not necessarily including the avulsion of a nerve root.

### 2.3. Outcomes

**Figure 1:**
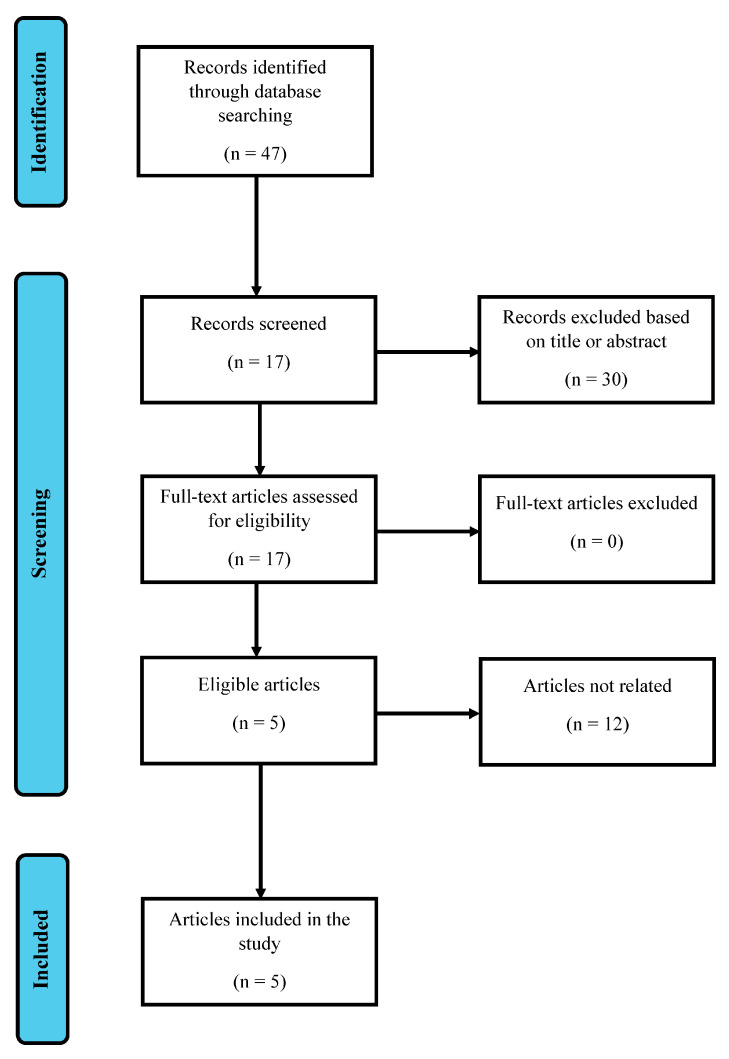
Article selection following the PRISMA^1^ guidelines.

A total of forty-six manuscripts were identified in the first phase or research, of which thirty were subsequently excluded based on the title and abstract. Further twelve articles were excluded based on the non-related topic covered. No articles were excluded due to the impossibility of either retrieving the full-text work or the language used was not English, leaving a total of five articles meeting the inclusion criteria. Included articles are reported in Table 1; the flowchart is reported in Figure 1.

## 3. Case Report

A 30-year-old man suffered a motorcycle incident in January 2023. Overall injuries accounted for a partial amputation of the right arm with a fracture of the humerus bone, multiple vertebral fractures without spinal cord involvement, ribs, and sternal fractures.

Once the acute phase was over, a complete paralysis of the right arm was observed. After excluding other potential causes, a diagnosis of a complete right brachial plexus roots avulsion was made.

The patient came to our attention in September 2023. A diagnosis of ulcerative colitis was the only other known pathology in his past medical history, in pharmacological treatment with Mesalazine. At the neurological examination he presented a 0/5 on the Medical Research Council (MRC) scale for muscle strength at the right hand, forearm, and arm; a winged scapula (scapula alata) on the right side; a 3/5 on the trapezius muscle; and 5/5 on the sternocleidomastoid muscle. No Hoffman-Tinel signs were elicitable. A palpable, soft, not painful mass over the collarbone was finally appreciable without sign of orthostatic headache.

**Figure 2:**
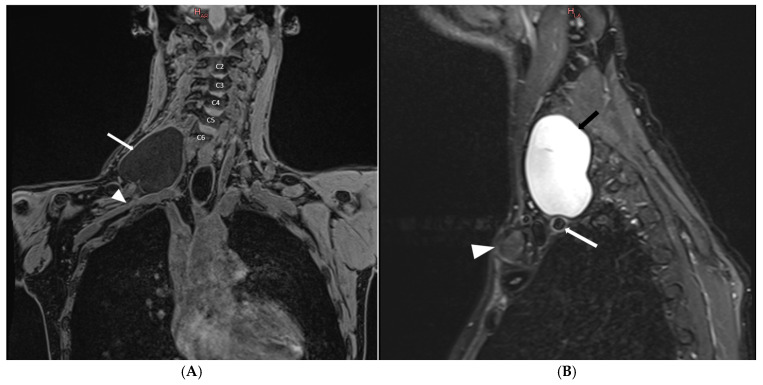
(**A**) T1-VIBE-weighted coronal-view MRI of right brachial plexus. A voluminous pseudomeningocele is shown (white arrow) with the highest point starting at C6 low endplate. The pseudomeningocele covers almost the entirety of the right brachial plexus and comes in contact with the subclavian artery (white arrowhead). (**B**) T2-STIR-weighted sagittal-view MRI. It is possible to appreciate the relationship of the pseudomeningocele (black arrow) with the subclavian artery (white arrow) and its localization in comparison with the clavicle (white arrowhead).

**Figure 3:**
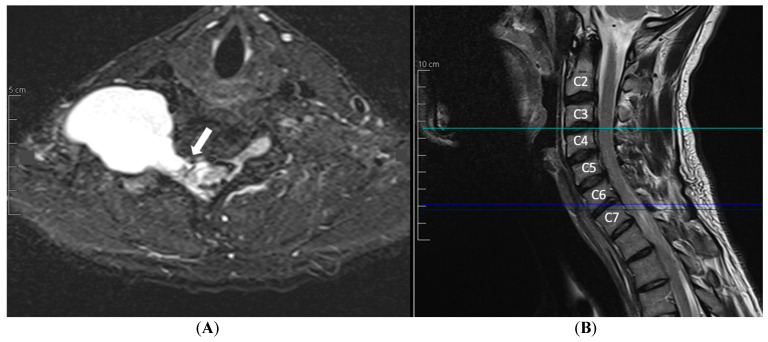
(**A**) T2-MEDIC-weigted axial view MRI showing the arachnoid fistula of the pseudomeningocele (white arrow), located at the neural foramen C6-C7, as shown in the (**B**) T2-weighted sagittal view MRI (middle blue line).

A plexus MRI of the right brachial plexus was carried out, revealing a C5-T1 roots avulsion together with a huge pseudomeningocele (62.5 × 62.2 mm) extending from the low endplate of C6 to the low endplate of T2 (Figure 2), with the fistula localized at neural foramen C6-C7 (Figure 3), while at the brain MRI no evidence of hydrocephalus nor intracranial hypotension was present.

A supraclavicular exploration of the right brachial plexus was proposed to perform an XI cranial nerve (Spinal Accessory nerve) to the Musculocutaneous nerve transfer for elbow flexion.

In January 2024, the patient was hospitalized in our department of Neurosurgery. During the planning phase, different options were considered for the control of the pseudomeningocele, such as a blood patch or a coil-assisted embolization, but were ultimately rejected because of the width of the fistula. After consultation with the Skull Base Unit (SBU) surgeons at our department, an External Lumbar Drainage (ELD) was chosen as a rescue method in case of rupture of the CSF collection. ELD was then positioned in a sterile fashion, with an initial drainage of about 20 mL of CSF; another 30 mL were drained during surgery.

A cutaneous incision was made along the deltopectoral groove and extended toward the base of the neck. The pseudomeningocele was immediately encountered. To avoid tearing it, the capsule was freed from the surrounding tissues with gentle blunt dissection. Puncture of the pseudomeningocele with an insulin pen needle was carried out to reach the fistula, with CSF removal of 50 mL. The cystic formation refilled almost immediately, and during its mobilization a small tear was created with immediate leakage of CSF, forcing the surgeon to rapidly pass a 0-non-absorbable thread around the fistula and underdoing a ligation of the fistula itself. CSF leakage stopped, the pseudomeningocele, now empty, was excised, and the injured brachial plexus was in reach.

The injured Musculocutaneous nerve was individualized, together with the Spinal Accessory nerve, the latter also with the help of intraoperative stimulation. Due to the wide gap between the two nerves, an autograft of about 5 cm of the right Sural nerve was utilized to connect the two nervous stumps. Finally, fibrin glue was used to protect the neurorrhaphies. In the closing phase, an abdominal fat patch was taken at the navel level and used as a cover of the pseudomeningocele ligature, reinforced with fibrin glue as a sealant. Whole operating time took 180 min.

At the end of surgery, during the awakening, a paralysis of the right hemidiaphragm was revealed, which did not cause any early harm to the patient. He was conducted back to the ward with the ELD closed and still in place with the right arm fixed through a Gilchrist bandaging.

In the evening of the same day, the ELD was reopened and set at 5 mL/h.

During hospitalization, the hemidiaphragm did not improve; the patient remained asymptomatic but required respiratory physiotherapy to avoid complications in the long run. The patient lay still in bed during the first day after surgery, always keeping the operated arm fixed and the ELD opened at the same fixed output of 5 mL/h.

Mobilization started from the second day, while the third day ELD was closed and removed the subsequent day.

The patient was discharged at home on the fifth day after surgery in good clinical condition, with the right arm fixed in place through the Gilchrist bandaging and with indications for follow-up. Passive mild physiotherapy was begun at 3 weeks from surgery with gradual regaining of complete range of movement of the right upper limb; at 2 months from surgery, electrical stimulation of the right upper limb muscles was begun. In the meantime, respiratory physiotherapy was conducted to improve respiratory function.

**Figure 4:**
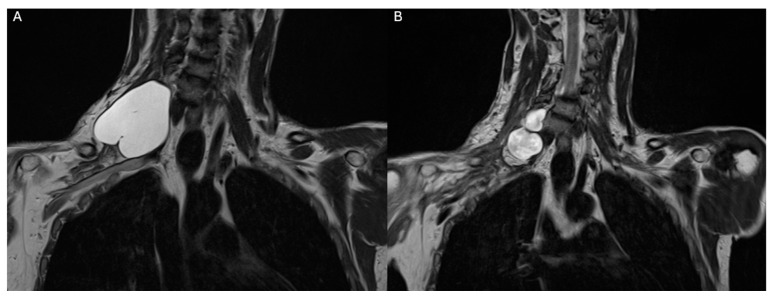
(**A**) T2-weighted coronal view of preoperative MRI of the pseudomeningocele, confronted with (**B**) the 6 months follow-up T2-weighted coronal view MRI showing a clear reduction in the pseudomeningocele’s dimension, not palpable at clinical inspection.

A late control MRI executed at 6 months showed a clear shrinking of the pseudomeningocele (Figure 4), which was not palpable anymore during the clinical evaluation.

At the last outpatient clinic visit at 6 months, a positive Hoffman-Tinel sign was experienced by the patient at the proximal third of the right arm. No active movement was evoked by the patient. No respiratory problems were recalled by the patient, and the wound had undergone satisfactory healing. A new outpatient clinic visit is scheduled for January 2025, 12 months from surgery.

## 4. Discussion

Post-Traumatic Pseudomeningoceles (PTPs) constitute a possible radiological finding after a spine trauma, especially when high energy is involved. They are commonly seen in conditions like AOD [10,11] with typical retropharyngeal pseudomeningoceles and nerve root avulsion. In the last case, brachial [12,13] or lumbar [7,14,15] plexus injuries, typically in pre-ganglionic lesion, lead to the formation of a steady leakage of CSF. Indeed, PTPs consist of a tear in the meningeal sheath around the nerve roots with extravasation of the CSF in the neighbouring paraspinous soft tissue at the neural foramen level [12], along the normal nerve course, more or less in communication with the spinal subarachnoid spaces, in what is called an arachnoid fistula. The subsequent enlargement of the PTP itself is favoured by the free and pulsatile flow of CSF [16]. Often considered indirect signs of preganglionic injury, their presence is a valuable but not pathognomonic sign of roots avulsion [12,13]; as a matter of fact, they are present in almost 80% of the avulsions, leaving a well 20% without pseudomeningocele formation [17]. Since they are filled with CSF, they are easily identifiable on T2-Weighted MRI [13], and normally they represent no more than a radiological finding at MRI. Nonetheless, in rare cases they might become symptomatic due to their size [7] or leading to complications such as a Spinal Cord Herniation (SCH) presenting with a Brown-Sequard syndrome [18,19,20,21]. Moreover, they might constitute an obstacle during a reconstructive surgery, as in the reported case. No clear guidelines nor standardized approach on the management of post-traumatic pseudomeningoceles after brachial/lumbar plexus injury are reported in the literature. This is because in most cases, the lack of symptoms or signs related to them does not require any surgical intervention. But in extremely rare cases, a strategy of treatment is required, and, to date, only five cases, to the best of our knowledge, have been reported in the literature. In three cases a direct approach to the lesion was opted for [5,9,16], while the other used either an endovascular-like approach [6] or a minimally invasive approach [8].

In the reported case, the high risk of rupture of the pseudomeningocele due to its volume and the subsequently high risk of a CSF leakage, a multidisciplinary consultation with the interventional neuroradiology team was undertaken; the option of a coiling-assisted embolization was rejected due to the width of the fistula at the C6-C7 foramen, while a blood patch was not suited due to the volume itself with a very low probability of the clot reaching the desired target. A second opinion with the surgeons of the SBU was therefore made due to the relatively high frequency of complications after a skull base surgery, in particular in the posterior fossa surgery, with CSF leakage and iatrogenic pseudomeningocele formation as major concerns [22]. The final decision was then the prophylactic placement of a closed continuous drainage of CSF via an ELD catheter with an initial intraoperative drainage of 20 mL and a following fixed output of 5 mL/h, adjustable if needed [23,24]. The rationale behind it was to create a low-resistance pathway for the CSF to avoid refilling the PTP. The placement itself did not come without risks, as infections and hemorrhage, considered negligible though in comparison to the risk of a post-operative CSF fistula; ELD was positioned before surgery in a sterile fashion, and prophylactic antibiotic therapy with Cefazoline was prolonged till the ELD removal. ELD remained in place and opened for a total of three days, considering the surgery day too. The first 24 h after surgery, the patient was not allowed to start mobilization to further decrease the risk of a replenishment of the CSF fistula, as is custom at our institution. Before removing it, a closure test of 24 h was carried out, with no leaking from the surgical wound nor collection of fluid masses under the skin at close palpatory inspection.

## 5. Limitations

The presented paper must be seen in light of several limitations, first and above all the scarcity of the pathology itself requiring a surgical approach; moreover, although ELD is frequently used in other fields to prevent CSF leakage, no assurance can be stated in this particular case for ELD to have played a key role. Finally, the duration of the placement of the CSF-drainage was arbitrary and based on subjective experiences.

## 6. Conclusions

In the literature, few solutions to manage the problem of a PTP are reported, and therefore it was decided to propose a possible strategy with the use of an external lumbar drainage, a solution already in use in other neurosurgical contexts with successful results in preventing CSF fistula or its relapse, especially in skull base surgery. To the best of our knowledge, this is the first reported case of its kind and therefore its major limitation, but we encourage the research in this field and the discussion about the presented topic.

## Figures and Tables

**Table 1:** Included articles of our systematic review ranging from November 1972 to May 2024.

Author(s)	Year	N° Patient (s)	Type of Management
Gass HH. et al. [5]	1972	1	Laminectomy with direct suturing of dural tears
Pascual-Gallego M. et al. [6]	2013	1	Direct puncture and injection of coils and onyx in the fistula
Rahimizadeh A. [7]	2020	1	Partial excision of the cyst wall and reinstitution of the root sleeve
Lad PB. et al. [8]	2021	1	Lumbar puncture with CSF drainage
Huang, Shih-Ting et al. [9]	2024	1	Direct suture of the dura mater and fibrin sealant along the suture line

## Data Availability

No new data were created or analyzed in this study. Data sharing is not applicable to this article.

## Abbreviations

PRISMA	Preferred Reporting Items for Systematic Reviews and Meta-Analyses;
AOD	Atlanto-Occipital Dislocation;
MRC	Medical Research Council;
MRI	Magnetic Resonance Imaging;
SBU	Skull Base Unit;
ELD	External Lumbar Drainage;
CSF	Cerebral Spinal Fluid;
PTP	Post-Traumatic Pseudomeningoceles;
SCH	Spinal Cord Herniation;
VIBE	Volumetric Interpolated Breath-hold Examination;
STIR	Short Tau Inversion Recovery;
MEDIC	Multiple Echo Recombined Gradient Echo.
